# Cryptotanshinone Inhibits the Growth of HCT116 Colorectal Cancer Cells Through Endoplasmic Reticulum Stress-Mediated Autophagy

**DOI:** 10.3389/fphar.2021.653232

**Published:** 2021-06-17

**Authors:** Xiaojing Fu, Wenwen Zhao, Kangkang Li, Jingyi Zhou, Xuehong Chen

**Affiliations:** ^1^ School of Basic Medicine, Qingdao University, Qingdao, China; ^2^ State Key Laboratory of Quality Research in Chinese Medicine, Institute of Chinese Medical Sciences, University of Macau, Macao, China

**Keywords:** cryptotanshinone, colorectal cancer, apoptosis, autophagy, endoplasmic reticulum stress

## Abstract

Among cancers, colorectal cancer (CRC) has one of the highest annual incidence and death rates. Considering severe adverse reactions associated with classical chemotherapy medications, traditional Chinese medicines have become potential drug candidates. In the current study, the effects of cryptotanshinone (CPT), a major component of *Salvia miltiorrhiza Bunge* (*Danshen*) on CRC and underlying mechanism were explored. First of all, data from *in vitro* experiments and *in vivo* zebrafish models indicated that CPT selectively inhibited the growth and proliferation of HCT116 and SW620 cells while had little effect on SW480 cells. Secondly, both ER stress and autophagy were associated with CRC viability regulation. Interestingly, ER stress inhibitor and autophagy inhibitor merely alleviated cytotoxic effects on HCT116 cells in response to CPT stimulation, while have little effect on SW620 cells. The significance of apoptosis, autophagy and ER stress were verified by clinical data from CRC patients. In summary, the current study has revealed the anti-cancer effects of CPT in CRC by activating autophagy signaling mediated by ER stress. CPT is a promising drug candidate for CRC treatment.

## Introduction

Nowadays, changes in human dietary structure and increase in life pressure has resulted in increasing incidence of colorectal cancer (CRC), especially in relatively younger age groups (Miller et al., 2020; Siegel et al., 2020). Clinically, the treatment of CRC commonly involves a combination of three classic strategies of oncology: chemotherapy, surgery, and radiation therapy. However, the approach of using chemotherapeutic drugs for CRC has several limitations, especially side-effect of drugs after repeated administrations (Roessler et al., 2010). Although significant advances have been made in our understanding of the molecular basis of this tumor type, novel efficacious therapeutic avenues are urgently needed.

Endoplasmic reticulum (ER) stress is a recognized factor in tumor growth (Schleicher et al., 2010). ER stress signaling, also known as unfolded protein response (UPR), is a cellular adaptation mechanism that occurs when endoplasmic reticulum homeostasis is destroyed following nutrient deprivation, hypoxia, or oxidative stress (Lu et al., 2014; Wang & Kaufman, 2016; Fang et al., 2021). The UPR is induced by three ER-anchored transmembrane receptors—inositol-requiring enzyme 1α(IRE1α), protein kinase RNA (PKR)-like ER kinase (PERK), and activating transcription factor-6 (ATF6), which detect misfolded proteins, expand ER protein folding capacity and decrease protein folding demand (Walter & Ron, 2011; Prasad & Greber, 2021). The UPR is an evolutionarily conserved stress response pathway tasked with reducing levels of unfolded/misfolded proteins and restoring ER homeostasis (Schleicher et al., 2010; Wang et al., 2021). If ER homeostasis cannot be restored, UPR drives the damaged or infected cells to apoptosis (Ferri & Kroemer, 2001; Huang et al., 2021; Li et al., 2021). When adaptive endoplasmic reticulum stress occurs, activation of UPR can induce protective autophagy and promote cell survival (Rouschop et al., 2010). However, it has been proven that triggering excessive stress on the persistent and severe endoplasmic reticulum can induce autophagy in tumor cells and ultimately lead to cell apoptosis (Rah et al., 2015; Xia et al., 2017; Fang et al., 2021; van Anken et al., 2021).

Autophagy is a process related to autophagy-related genes (ATG), which transports endogenous or exogenous cytoplasmic substances to lysosomes for degradation (Mokarram et al., 2019; Zhao et al., 2021). Thecytoplasmic form of LC3 (LC3-I) is conjured with phosphatidylethanolamine (PE) to form the LC3-phosphatidylethanolamine conjugation (LC3-II), which is known as a hallmark of autophagy (Kang et al., 2019; Xie et al., 2020). Beclin-1 plays an important role in autophagy initiation, which not only affects every link of autophagy, but also plays a key role in the regulation of the interaction between autophagy and apoptosis (Kang et al., 2011; Noguchi et al., 2020). It has been reported that the some chemotherapeutic drug-stimulated autophagy pathway can activate caspase-3, thereby inducing the apoptosis of tumor cells (Mowers et al., 2018). At same times, studies have confirmed that cell death in CRC can be achieved by inducing autophagy-dependent apoptosis pathways (Pedro et al., 2015; Grasso et al., 2016; Nagappan et al., 2017; Xia et al., 2017; Maranhão et al., 2020).

Herbs have recently aroused more and more interests in the discovery of anticancer therapies because they have long been used as alternative therapies for various diseases, including cancer, coronary heart disease, and diabetes, with relatively fewer side effects (Zhao et al., 2019; He et al., 2020; Sun et al., 2020; Zhao et al., 2020; Zheng et al., 2020). Multiple studies have reported that some active ingredients of *Salvia miltiorrhiza Bung*e (*Danshen*) have anti-tumor activities, including hepatoma, and prostatic cancer (Xu et al., 2012; Luo et al., 2020; Wang et al., 2020). Cryptotanshinone (CPT) is one important active ingredient from *Danshen* and is usually used to treat atherosclerosis, alzheimer’s disease, hyperlipidemia, liver fibrosis, chronic renal failure, and gynecological diseases in Asian countries, with few reported serious side effects (Xu et al., 2012; Cai et al., 2016; Ding et al., 2016; Liu et al., 2020). Currently, many researchers are investigating CPT and have reported that CPT exhibits direct cytotoxic effects on multiple types of cancer cells. while exact mechanism is yet to be elucidated. In the current study, effects of CPT on CRC cells and underlying mechanism were explored.

## Materials and Methods

### Materials

RPMI1640 (Jinuo, Jiangsu, China); Fetal bovine serum (BiologicalIndustrie, Israel); ER Stress Antibody Sampler Kit (CST, United States); ATF-4; Caspase-3 (CST, United States); Cleaved caspase-3(CST, United States); LC3B (Zenbio, Chengdu, China); Anti-mouse (CST, United States); Anti-rabbit (CST, United States); JC-1 (Beyotime, Shanghai, China); LDH Cytotoxicity Assay Kit (Beyotime, Shanghai, China); Autophagy Antibody Sampler Kit (CST,United States); FITC Annexin Ⅴ Apoptosis Detection Kit (BD, US); Sodium 4-phenylbutyrate (4-PBA) (Bidepharm, Shanghai, China); Spautin-1; 4% Paraformaldehyde (4% PFA) (Solarbio, Beijing, China); 0.5% Crystal violet (Solarbio, Beijing, China); spautin-1; Immunohistochemistry (IHC) detection system kit (Rabbit) (Bioss, Beijing, China); Cryptotanshinone (Herbest, Shanxi, China); 0.003% tricaine (Sigma, United States); DiI (Invitrogen, Carlsbad, CA, United States); Nanoliter Injector (Drummond Scientific Company, Broomall, PA); Cisplatin Injection (Xinnuo, Jiangsu, China); dimethyl sulfoxide (DMSO) (Solarbio, Beijing, China); RIPA buffer (Solarbio, Beijing, China); Acridine orange detection kit (Leagene, Beijing, China).

### Cell Culture

CRC cells (HCT116, SW480, and Sw620) were cultured in RPMI1640 supplemented with 10% FBS and 1% penicillin/streptomycin at 37°C in a humidified atmosphere with 5% CO_2_.

### Clinical Samples

The procedures in the current study has been formally approved by the ethics committee of Qingdao University (Qingdao, China) and written consent forms were approved by all patients. In this study, tumor tissues were collected from 56 patients with colorectal cancer who underwent surgery between June 2019 and February 2020. Diagnosis and staging were carried out by 2 independent senior oncologists blinded to the data. Colorectal cancer tissues and adjacent non-cancer tissues were collected and stored at −80°C until further uses.

### MTT Assay

Cells at a density of 5 × 10^3^ per well were seeded in 96-well plates, treated for 24 or 48 h, and then the viability of treated cells was determined with the MTT assay according to the supplier’s instructions.

### Colony-Forming Assay

The cells were seeded in a 6-well plate at a density of 600 cells/well for 24 h, and then stimulated with drugs for 8 h. After the treatment, incubation was continued for 14 days in a complete culture medium at 37°C and 5% humidified CO_2_. At the end of incubation, the cells were washed twice with PBS, fixed with methanol for 15 min, and stained with 0.5% crystal violet for 15 min at room temperature. A colony is defined as accumulation of at least 50 cells. The visible colonies were counted, and the colony formation rate was calculated with the following equation:

Colony formation rate=(number of colonies/number of inoculated cells)×100%.

### Lactate Dehydrogenase Cytotoxicity Assay

LDH assay was used to detect cytotoxicity following different treatments, using a LDH assay kit. Studies were performed following the instructions provided by the manufacturer.

### Flow Cytometry Assay

After drug treatment, the cells were washed with PBS, and digested with trypsin. After the cells were collected, they were washed with PBS for 2 times and centrifuged. The cells were resuspended with Annexin Ⅴ-FITC binding buffer; Add Annexin Ⅴ-FITC to the cell suspension and incubate for 15 min in the dark The PI was added, and the cells were filtered and detected by flow cytometry. Data were analyzed with the CytExpert software (Beckman Coulte, United States).

### Western Blotting Analysis

Wash cells with precooled PBS. RIPA cell lysate is used to lyse cells. After the protein was heated and denaturated, 15–30 μg of protein was taken from each sample for protein electrophoresis. After membrane transfer, the PVDF membrane was sealed with 5% skimmed milk at room temperature for 60 min. Then, they were incubated overnight in a buffer containing the antibodies at 4°C. Wash 3 times with PBST (PBS with 0.1% Tween-20), and the corresponding HRP-linked Antibody was selected and incubated at room temperature for 1 h. After washing again with PBS for 3 times, drop ECL chemiluminescence solution. Photographs were taken in a chemiluminescence imager. Densitometry analysis was performed with Image J software (NIH, United States).

### JC-1 Assay

Cells at 5 × 10^3^ per well were seeded in 96-well plates, treated with different concentrations of CPT for 48 h. The cells were washed twice with warm PBS and incubated with JC-1 (2 µM final concentration) for 30 min in the dark. After two more washes with PBS, images were captured with an fluorescence microscope. Densitometry analysis was performed with Image J software (NIH, United States).

### Acridine Orange Staining

Cells were cultured in a 24-well plate at a density of 5–10 × 104 per well overnight; treatments were then performed as described in triplicate for 48 h. Then the supernatant was aspired, cells were rinsed with PBS for three times, and then 0.5 ml of 0.1 mg/ml acridine orange working solution was added to each well. Cells were incubated in a 5% CO_2_, 37°C constant temperature incubator for 15 min (avoid light), and then the supernatant was aspired, pictures were taken with an inverted fluorescence microscope after three washes with PBS.

### Immunohistochemistry Analysis

Continuous sections were acquired from formalin fixed, paraffin-embedded tumor tissues, dewaxed in xylene, rehydrated in gradient ethanol, immersed in deionized water, and heated in 0.01 M sodium citrate antigen-repair buffer in microwave oven for 15 min. The Sections were then treated with 3% hydrogen peroxide in methanol (endogenous peroxidase blocker) for 30 min, and then blocked with 5% bovine serum albumin (BSA) to block non-specific binding for 15 min. Place in PBS buffer for 10 min and repeat 3 times. Then the sections were incubated with primary antibody (BIP [1:200], LC3B [1:100], cleaved caspase-3 [1:250]) overnight at 4°C. After incubation with a secondary antibody, 3, 3’-diaminobenzidine tetrachloride was used to visualize the stainings. After counterstaining with hematoxylin, sections were dehydrated in ethanol, cleared in xylene, and sealed with resin. The sections were photographed under a microscope and analyzed with Image J software (NIH, United States).

### *In Vivo* Tumor Growth Model

At the night before injection, female and male zebrafishes were matched at a ratio of 1:2 and placed in the mating aquarium; 2 h after fertilization, the zebrafishes were treated with PTU (1-phenyl-2-thiourea, 0.2 mM) for lucidification. HCT116 and SW620 cells were labeled with DiI. 48 2 hpf zebrafish were anesthetized with 0.003% tricaine. Then microinjections of the cell suspensions into the yolk sac was performed on 48 2 hpf zebrafish embryos with Nanoject II automatic booster syringe. Carbon dioxide injection was used to ensure the 5 nL injection volume. After receiving corresponding treatments, the animals were kept for 3 days. At 0 and 3 days after injection, animals were anesthetized with 70% ethanol, and then subjected to fluorescence microscopy. All experiments were approved by the Animal Research Ethics Committee of the University of Qingdao.

### Statistical Analysis

Data were expressed as the means ± SD from three independent experiments. Statistics were performed with Prism 5.0 statistical analysis software. After normality tests, the mean differences of groups were assessed with one way analysis of variance (one-way ANOVA), followed by post hoc Student Newman-Keuls test. All statistical tests were two-sided, and *p* < 0.05 was considered to be significant. Calculated *p*-values of *p* < 0.05, *p* < 0.01, and *p* < 0.001 were as indicated.

## Results

### Cryptotanshinone Treatment Selectively Inhibited CRC Cells Proliferation

**FIGURE 1:**
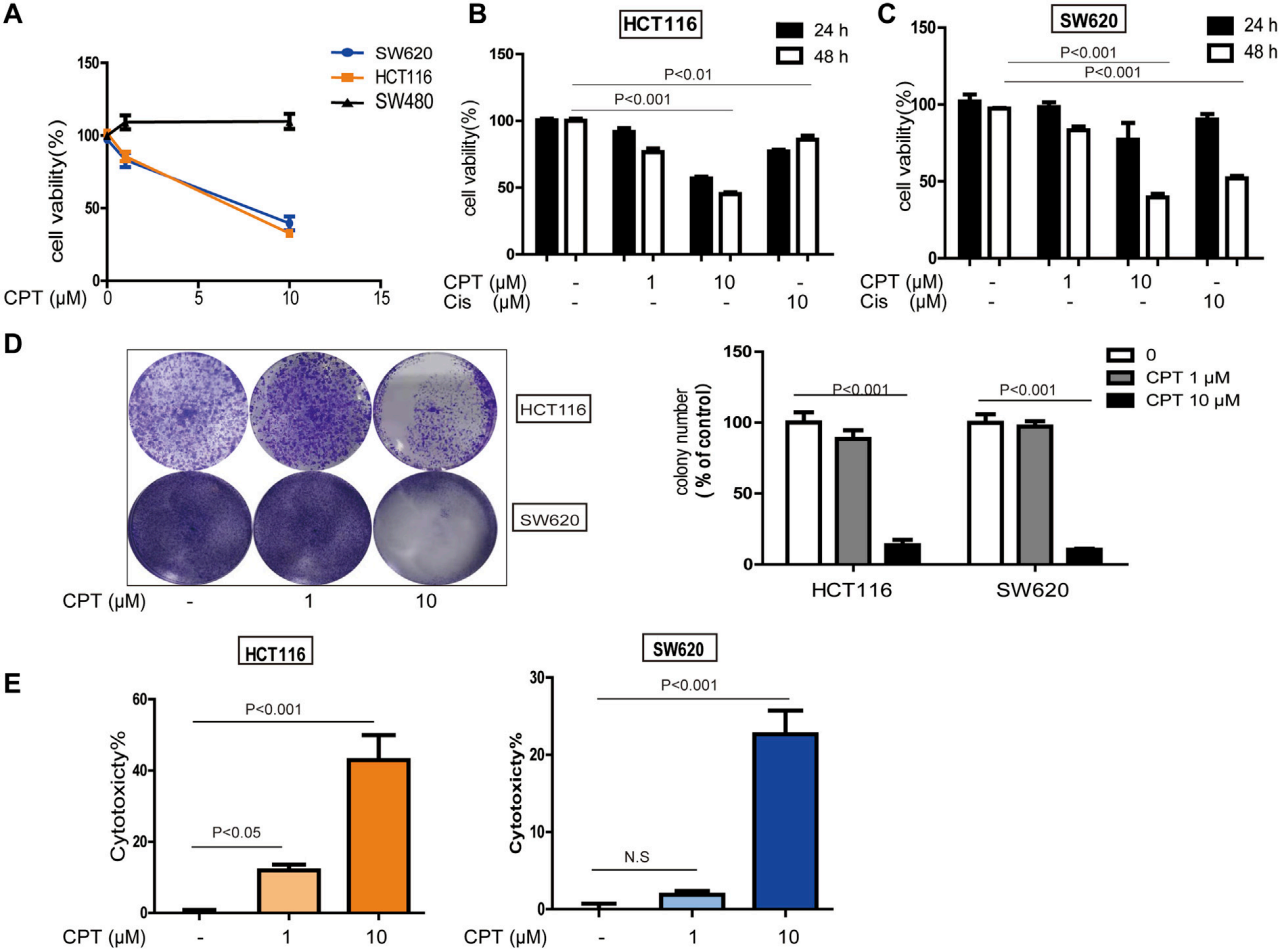
The role of CPT against colorectal cancer. MTT assay **(A)** was used to measure the activity of SW480, HCT116, and SW620 cells treated with concentrations of CPT for 48 h. SW620 and HCT116 were cultured in different concentrations of CPT or cisplatin (Cis) for 24 or 48 h, respectively, and cell proliferation rates were analyzed by MTT assay **(B,C)**. Colony formation assay of SW620 and HCT116 cells treated with the indicated concentrations of CPT. Representative images **(Left)** and quantification of colonies **(Right)** were shown **(D)**. HCT116 cells and SW620 cells were treated with different concentrations of CPT for 48 h, and the release amount of lactate dehydrogenase was analyzed by LDH assay, and the cytotoxicity was calculated **(E,F)**. CPT, cryptotanshinone; LDH, lactate dehydrogenase; Cis, cisplatin.

The incidence of colorectal cancer is among the top three worldwide (Siegel et al., 2020a; Siegel et al., 2020). CPT exhibited potent cytotoxicity on a series of CRC cells while underlying mechanisms are unclear. In the current study, three main types of CRC cells (HCT116, SW620, and SW480) were treated with CPT. As shown in Figures 1A–C, CPT treatment significantly inhibited SW620 and HCT116 cells proliferation and growth in a dose and time- dependent manner (optimum stimulation time:48 h; stimulation dose:10 μM), while no remarkable cytotoxicity were observed on SW480 cells. Additionally, the results of LDH assay and colony-forming assay in both HCT116 and SW620 cells further indicated that CPT treatment selectively induced cytotoxicity in HCT116 and SW620 cells (Figures 1D–F).

### Cryptotanshinone Treatment Inhibited HCT116 and SW620 Tumor Growth in Zebrafish Models

**FIGURE 2:**
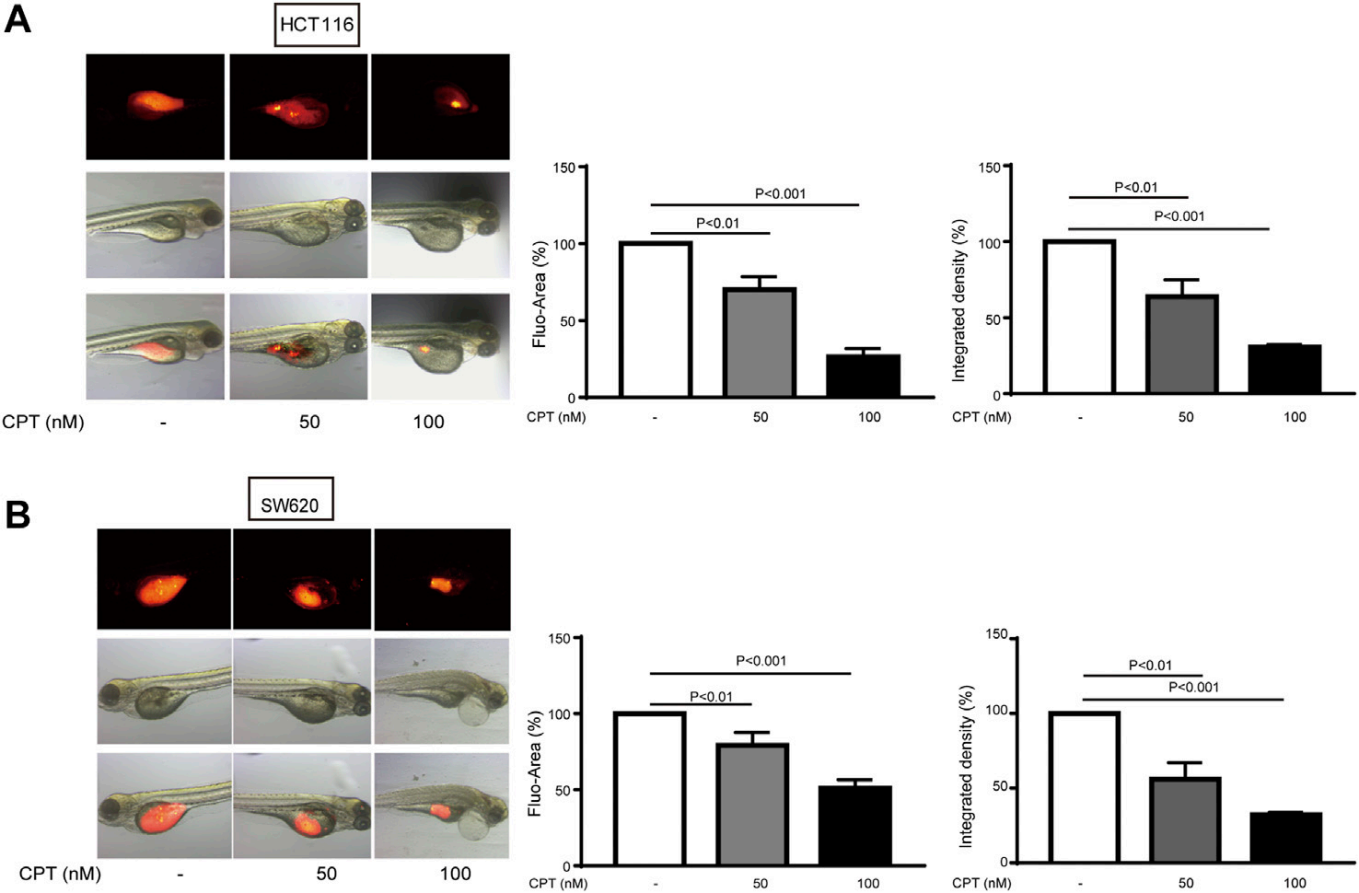
The inhibitory effects of different concentrations of CPT on tumor growth of HCT116 and SW620 zebrafish models were determined. The inhibitory effects of different concentrations of CPT on tumor growth of HCT116 and SW620 zebrafish were determined. The tumor was indicated by red fluorescence **(A)**, calculated as a percentage of the control value (*n* = 8) **(B)**. CPT, cryptotanshinone.

To further verify the data from *in vitro* experiments, xenograft zebrafish model was established to simulate the internal environment to evaluate the anti-tumor effects of CPT. As shown in Figure 2, the growth of the tumor formed with either HCT116 (Figure 2A) or SW620 (Figure 2B) was significantly inhibited after 3 days of CPT treatment at a concentration of 50, 100 nM, which was consistent with the data from *in vitro* experiments.

### Cryptotanshinone Induced HCT116 and SW620 Cells Apoptosis

**FIGURE 3:**
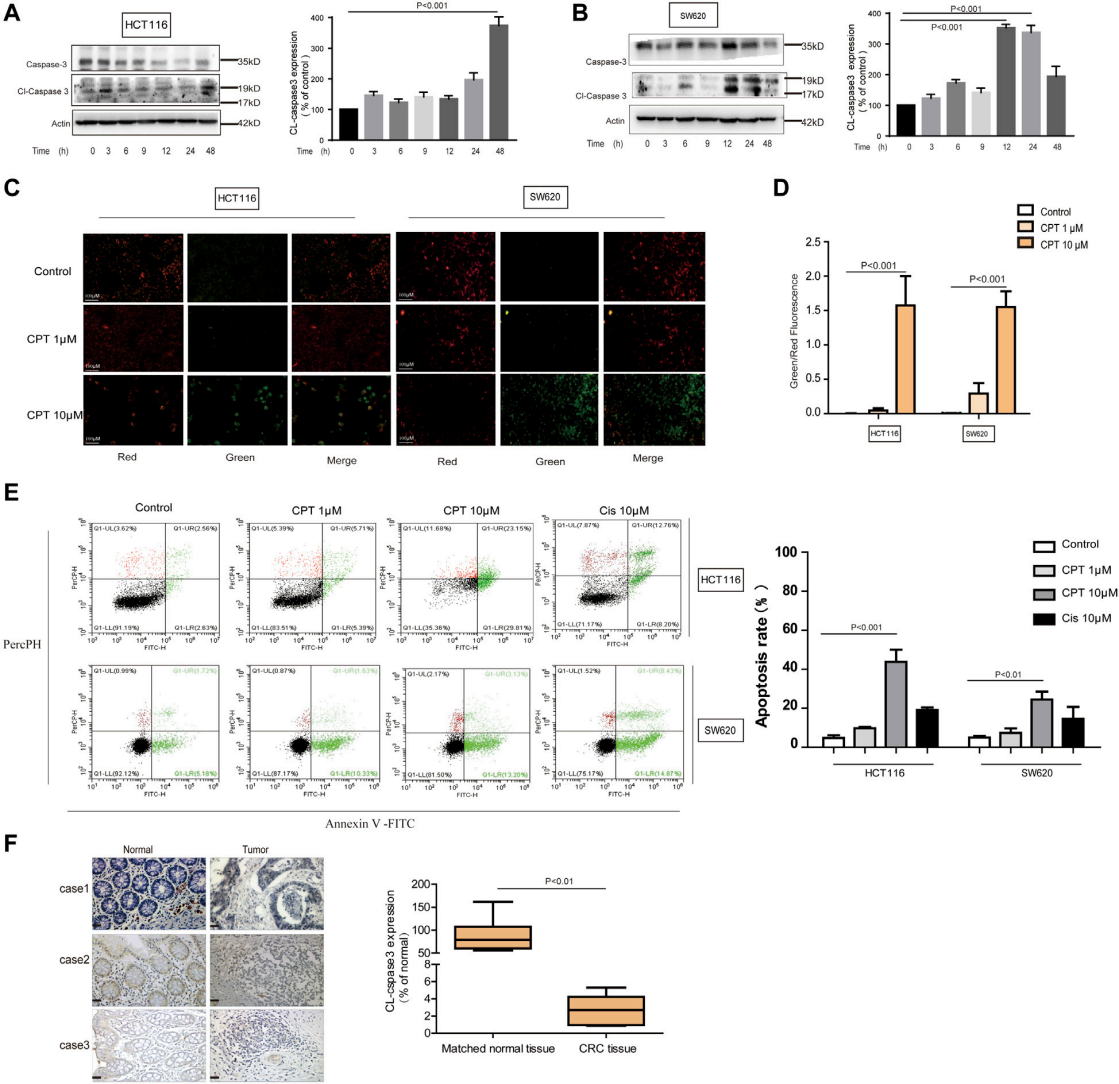
CPT can induce apoptosis of CRC cells. Caspase 3 and cleaved caspase3 in the lysates of HCT116 and SW620 cells treated with CPT (10 μM) for different time were detected by western blot assay and quantified by Image J **(A,B)**. The JC-1 assay was used to detect HCT116 cells and SW620 cells treated with different concentrations of CPT for 48 h. The change of red or green color was observed under fluorescence microscope (200 ×) **(C)** and green/red was calculated **(D)** (*n* = 3). Apoptosis of HCT116 cells and SW620 cells treated as in (C) was determined by Annexin Ⅴ-FITC/PI staining **(E)**. The immunohistochemical analysis of the clinical samples were performed, and the expression of cleaved caspase3 was detected in the tumor tissue and the corresponding para-cancer tissue, and the quantification analysis was performed with Image J **(F)**. CPT, cryptotanshinone; CRC, colorectal cancer.

Exact mechanisms of CPT-induced CRC cell death remain unknown, and were further explored in the current study. To examine whether apoptosis is associated with the anti-cancer effect of CPT, we used JC-1 staining to detect changes in mitochondria, Western blotting analysis to detect apoptosis-related proteins, and flow cytometry to evaluate the apoptotic ratio using flow cytometry. As shown in Figures 3A–G, CPT treatment significantly induced apoptosis in CRC cells. Increased cleaved caspase-3 expression levels were observed in CPT-treated CRC cells and maximized at 48 h in HCT116 cells and at 12 h in SW620 cells (Figures 3A,B). Besides, based on JC-1 assay, much more green fluorescence was observed in CPT-treated cells and green/red ratio were remarkably increased in cells treated with CPT, indicating severe mitochondrial destruction (Figures 3C,D). IHC was used to detect clinical samples, and the results showed that the expression of cleaved caspase3 in colorectal cancer tissues was significantly lower than that in non-cancerous tissues (Figure 3F). Collectively, CPT inhibited CRC growth by inducing apoptosis accompanied with mitochondrial dysfunction.

### Cryptotanshinone Promoted HCT116 Cells Apoptosis via Autophagy, Which Was Suppressed in CRC Tissues From Patients

**FIGURE 4:**
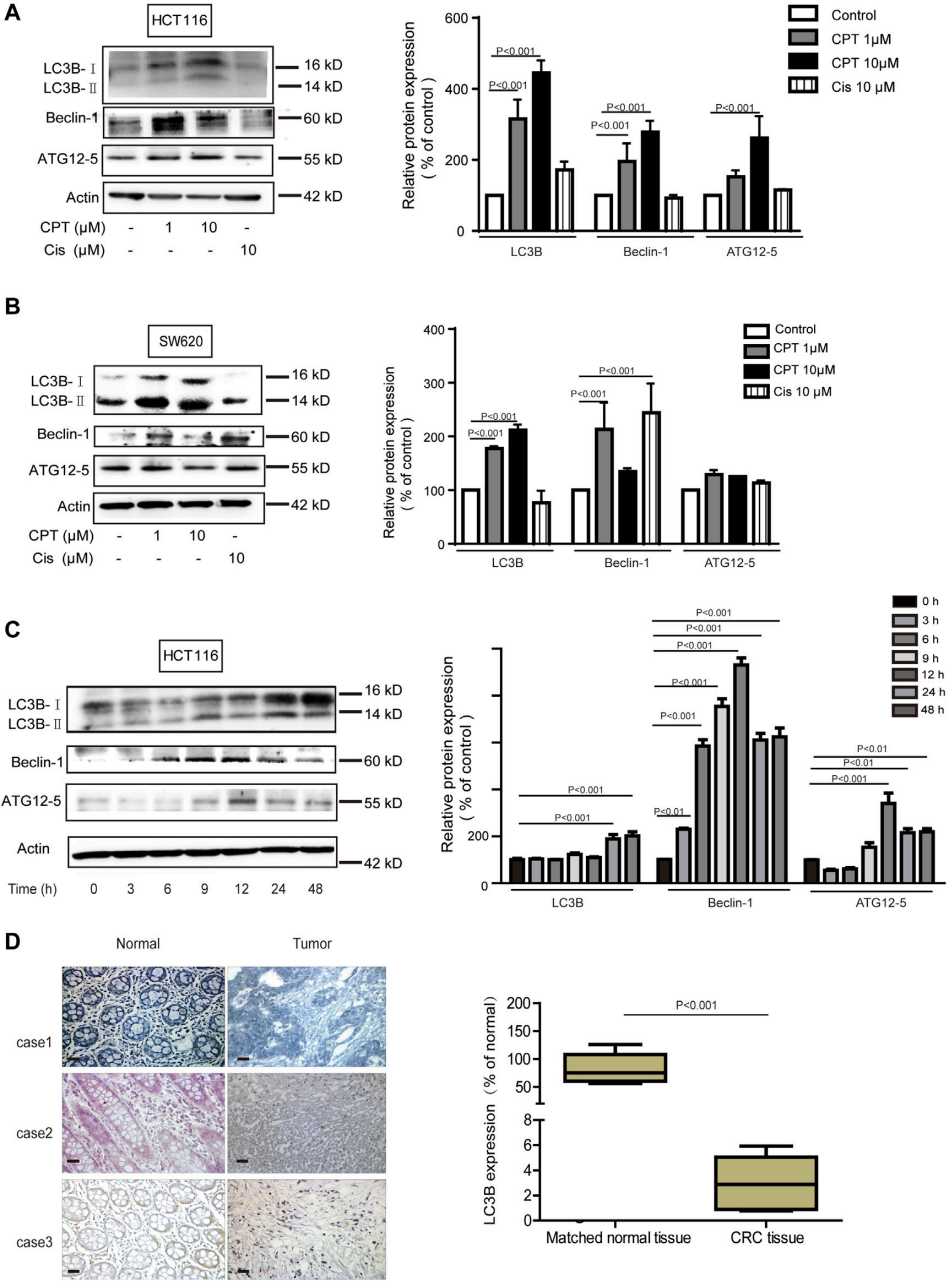
In CRC cells, autophagy mediates the anti-tumor effect of CPT. LC3B, Beclin-1, and ATG12–5 of HCT116 cells **(A)** and SW620 cells **(B)** treated with different concentrations of CPT or Cis for 48 h was detected by immunoblotting and quantitatively analyzed by ImageJ. LC3B, Beclin-1, and ATG12–5 in HCT116 cells treated with CPT at a concentration of 10 μM for different times was detected by western blot and quantitatively analyzed by Image J **(C)**. The immunohistochemical analysis of the clinical samples were performed, and the expression of LC3 was detected in the tumor tissue and the corresponding para-cancer tissue, and the quantification analysis was performed with Image J **(D)**. CPT, cryptotanshinone; CRC, colorectal cancer.

**FIGURE 5:**
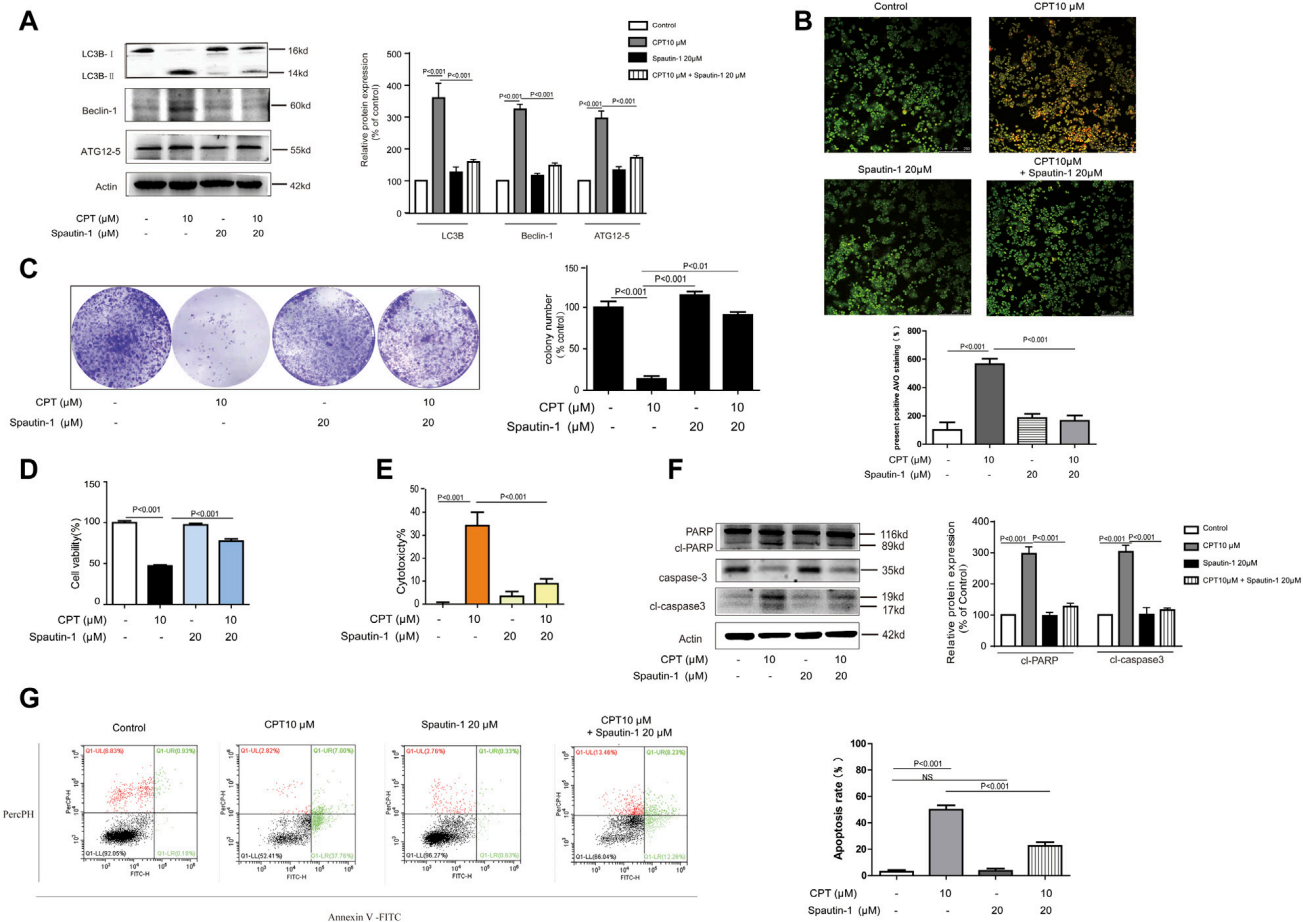
CPT can induce autophagy - mediated apoptosis in CRC cells. HCT116 cells were treated with or without CPT (10 μM) for 48 h in culture medium in the presence or absence of spautin-1 (20 μM). The expression of autophagy-related proteins (ATG12–5, Beclin-1, LC3B) in cell lysate was measured by western blot **(A)**. Fluorescence microscopy (200 ×) following AO staining detected the number of autophagosomes in HCT116 cells treated as in (A), and quantitative analysis was performed by ImageJ **(B)**. In the presence or absence of spautin-1 (20 μM), the colony formation assay of HCT116 cells treated with or without 10 μM CPT was performed, and the left image was representative and the right image was quantitative **(C)**. MTT assay **(D)**, and LDH release **(E)** assay were used to determine HCT116 cells treated as in **(C)**. Caspase-3 and PARP in HCT116 cells treated as in **(A)** were analyzed by western blotting assay and quantitatively analyzed by ImageJ **(F)**. Apoptosis of HCT116 cells treated as in **(C)** was quantified by flow cytometry **(G)**. CPT, cryptotanshinone; LDH, lactate dehydrogenase; CRC, colorectal cancer; AO, acridine orang.

Growing evidence has indicated that autophagy is closely linked with apoptosis induction (Jhou et al., 2020). Meanwhile CPT is considered as an important inducer of autophagy (Xu et al., 2017). In the current study, expression levels of autophagy-related proteins were increased with CPT treatment in a time- and concentration-dependent manner in HCT116 cells but not in SW620 cells, suggesting that CPT induced autophagy to regulate HCT116 cells viability (Figures 4A–C). To further explore the role of autophagy in the biological process of HCT116 cells, impacts of autophagy inhibitor treatment on CPT-induced cytotoxicity was tested on HCT116 cells (Figures 5A,B). As shown in Figure 5C–E, autophagy inhibitor Spautin-1 effectively ameliorated CPT-induced cytotoxicity in HCT116 cells. In addition, our study also confirmed that spautin-1 can effectively inhibit CPT induced apoptosis (Figures 5F,G). Therefore, the induction of HCT116 cell death by CPT is likely mediated via autophagy mediated apoptosis. In the clinical samples, the results from IHC staining showed that the expression levels of LC3B were remarkably higher while those of cleaved caspase 3 were significantly lower in CRC tissues relative to matched non-cancerous tissues (Figures 3F, 4D). Above data showed that autophagy was the potential target for CPT in CRC treatment.

### Cryptotanshinone Monitored HCT116 Cells Apoptosis Mediated by Endoplasmic Reticulum Stress Which Was Lowly Expressed in CRC Tissues From Patients

**FIGURE 6:**
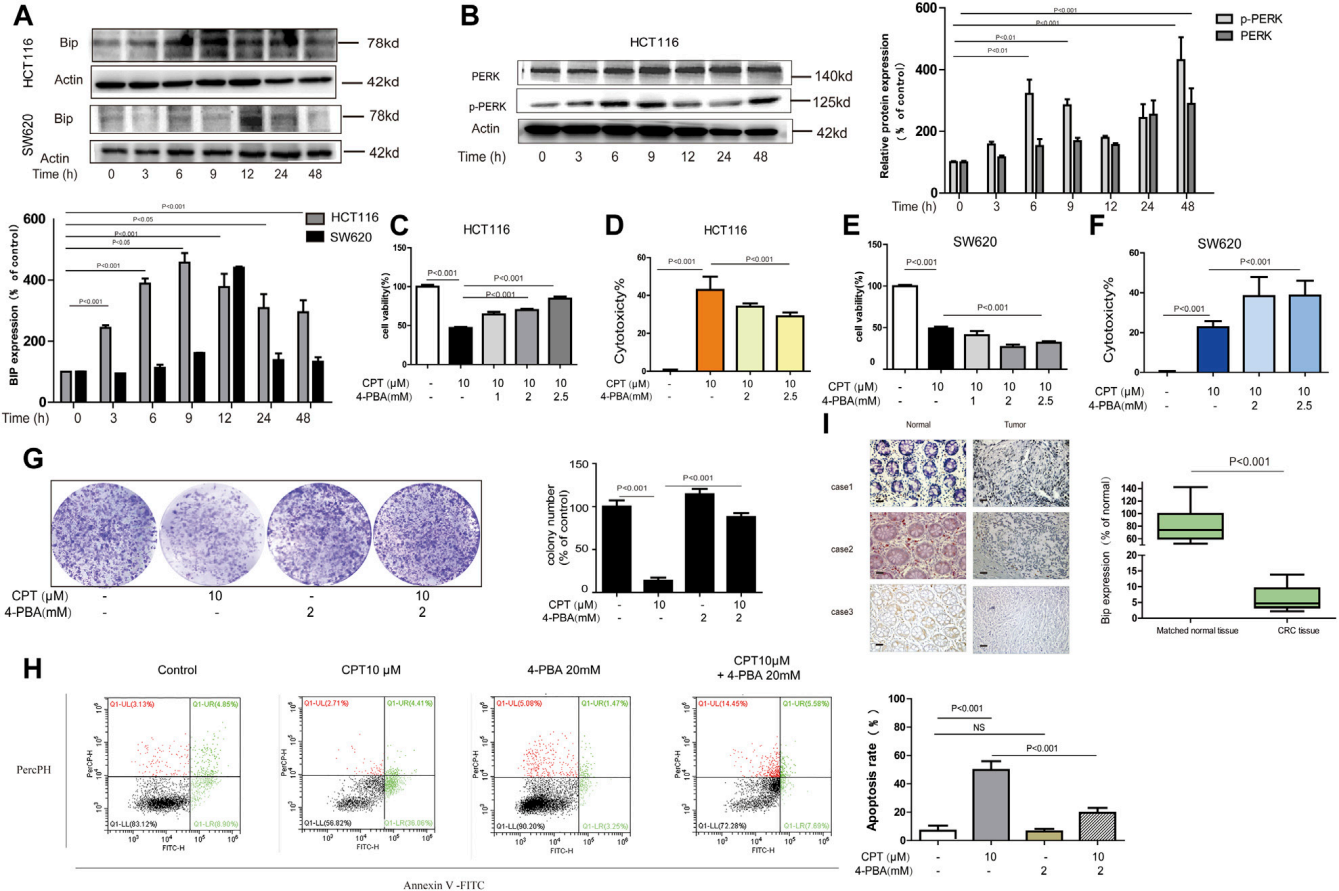
In HCT116 cell, ER stress mediates the anti-tumor effect of CPT. Western blot was used to determine BIP in HCT116 cells and SW620 cell lysates treated with CPT (10 μM) for different times **(A)**. PERK and p-PERK was detected in HCT116 cells treated as in (A) using western blotting analysis **(B)**. In the presence or absence of 4-PBA (1, 2, and 2.5 mM), HCT116 cells, and SW620 cells treated or untreated with CPT (10 μM) were cultured for 48 h and tested by MTT assay **(C, E)** and LDH assay **(D, F)**. In the presence or absence of 4-PBA (2 mM), the colony formation assay of HCT116 cells treated with or without CPT (10 μM) was performed and quantified with Image J **(G)**. Apoptosis of HCT116 cells treated as in **(G)** was detected by flow cytometry **(H)**. The immunohistochemical analysis of the clinical samples were performed, and the expression of BIP was detected in the tumor tissue and the corresponding para-cancer tissue, and the quantification analysis was performed with Image J **(I)**. CPT, cryptotanshinone; ER stress, endoplasmic reticulum stress; LDH, lactate dehydrogenase.

It is generally accepted that the generation of endoplasmic reticulum stress plays an important role in apoptosis by changing the internal environment of cells (Alper et al., 2020) (Wu et al., 2020). Recent studies reported that CPT is a crucial ER stress promoter in some colorectal cancers (Wang L. et al., 2020). Therefore, the roles of endoplasmic reticulum stress in growth of HCT116 and SW620 were explored in the current study. First of all, overexpression of ER stress protein was observed in both HCT116 and SW620 cells exposed to CPT (Figures 6A,B), which was further confirmed by the IHC results from human colon cancer tissues (Figure 6I). Next, based on results of MTT assay, LDH assay and plate cloning assay, ER stress inhibitor 4-PBA significantly alleviated CPT-induced damages on HCT116 cells, while no remarkable effects were observed on SW620 cells. Such results further revealed the specific selectivity of CPT on CRC cells and this regulation is likely mediated via ER stress (Figures 6C–G). Furthermore, apoptosis induced by CPT could be abolished by 4-PBA treatment, which indicated that CPT induced ER stress could induce apoptosis on HCT116 cells (Figure 6H).

### Endoplasmic Reticulum Stress Lied Upstream of Autophagy Leading to HCT116 Cells Death in Response to CPT Treatment

**FIGURE 7:**
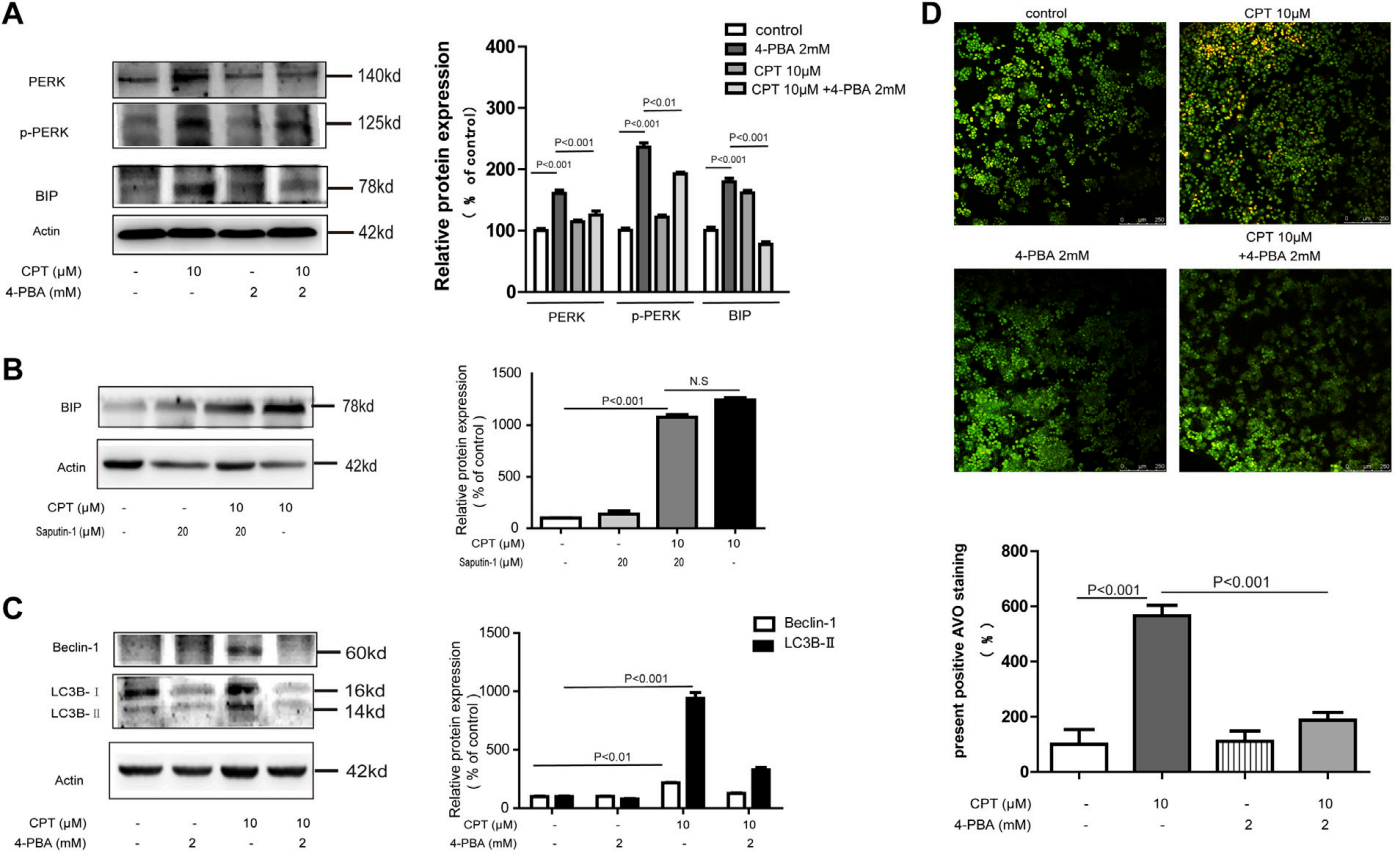
CPT induced ER stress mediated autophagy in HCT116 cells. Expression levels of PERK and P-PERK in HCT116 cells treated with or without 4-PBA (2 mM) in the presence or absence of CPT (10 μM) **(A)**. HCT116 cells treated with or without Spautin-1 (20 μM) for 48 h were treated with or without CPT (10 μM). The expression of BIP in the lysate was determined by western blot and quantified by Image J **(B)**. To test for Beclin-1 and LC3 expression in 10 μM CPT processed or untreated HCT116 cells, with or without 4-PBA (2 mM) **(C)**. Under fluorescence microscope (200 ×), the number of autophagosomes in HCT116 cells was stained by AO staining and quantified by ImageJ software **(D)**. CPT, cryptotanshinone; ER stress, endoplasmic reticulum stress; AO, acridine orang.

In the current study, both ER stress and autophagy were shown to be involved in the apoptotic process of HCT116 cells in response to CPT treatment while the association between them still needs to be explored. As shown in Figure 7A,B,D, ER stress inhibitor 4-PBA effectively inhibited the expression of autophagy-related proteins such as Beclin-1 and LC3B in CPT-treated HCT116 cells. However, autophagy inhibitor spautin-1 had little effects on the expression of BIP. We speculated that in HCT116 cells, ER stress is located upstream of autophagy pathway in damaged HCT116 cells (Figure 7C). Our results showed that autophagy induced by CPT could be inhibited by 4-PBA, which was subsequently verified by Acridine orange staining.

## Discussion

CPT is a major component derived from *Salvia miltiorrhiza* (Xu et al., 2017; Chen et al., 2020; Liu et al., 2020). Nowadays, multiple studies have confirmed that CPT has inhibitory effect on a variety of cancer cells, such as liver cancer, non-small cell lung cancer, breast cancer, and ovarian cancer (Chen et al., 2014; Yang et al., 2018; Qi et al., 2019; Li et al., 2020; Luo et al., 2020). However, its effects on colorectal cancer is poorly studied. In the current study, CPT exhibited obvious cytotoxicity on both HCT116 and SW620 cells while have no remarkable effects on SW480 cells, suggesting the selectivity of CPT treatment on CRC cells.

Apoptosis is known as type I programmed cell death characterized by chromatin condensation, DNA fragmentation, and the formation of apoptotic bodies (Xie et al., 2020). Various pathways initiated by apoptosis lead to the cleavage of caspase-3, which leads to irreversible cell death (Xu et al., 2017; Xie et al., 2020). It has been reported by Xu *et al* that CPT induced the apoptosis in multidrug-resistant human CRC cells SW620 Ad300 (Xu et al., 2017). Consistent with this result, our research results show that the treatment of CPT can induce apoptosis of CRC cells HCT116 and SW620. Moreover, caspase-dependent pathway was activated and mitochondrial dysfunction occurred in response to CPT treatment in both HCT116 and SW620 cells, indicating cytotoxicity in a caspase-dependent manner.

Although autophagy is well recognized as a cell survival process that promotes tumor development, it can also participate in programmed cell death independent of caspase (Rouschop et al., 2010; Denton and Kumar, 2019; Gómez-Díaz et al., 2019). In the current study, autophagy occurrence was detected in CRC cells treated with CPT (Park et al., 2014; Wang et al., 2020). Interestingly, autophagy inhibitor selectively prevented HCT116 cells death while have negative effects on SW620 cells. It has been reported that emodin leads to apoptosis of colon cancer cells through the oxidative stress pathway in an autophagy-dependent manner (Wang et al., 2018). In the present study, our findings indicated that inhibition of autophagy can prevent the apoptosis of HCT116 cells induced by CPT.

**FIGURE 8:**
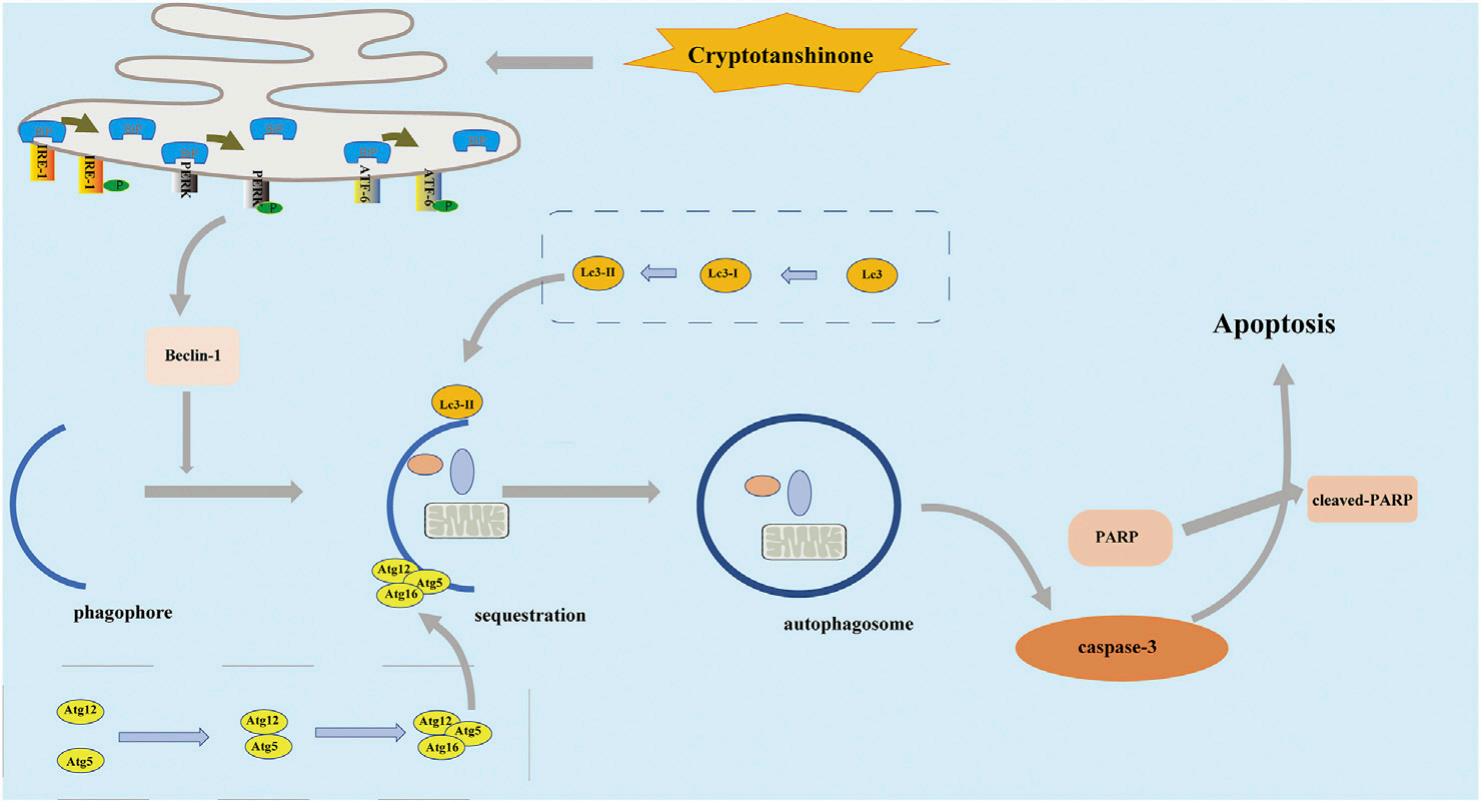
In the treatment of HCT116 cells by CPT, the apoptosis pathway is activated, which is associated with endoplasmic reticulum stress, and autophagy. CPT, cryptotanshinone.

ER stress is considered a common feature in various types of blood and solid cancers (Bhardwaj et al., 2019; Xia et al., 2021). In the current study, ER stress participation was observed in the growth and proliferation of several solid tumors, including CRC cells. Furthermore, ER stress inhibitor selectively attenuated CPT-induced cytotoxicity on HCT116, suggesting the significance of ER stress in CRC cell death. Meanwhile, ER stress inhibitor had little effects on SW620 cells. It is possible that CPT induced SW620 cell death by unknown alternative mechanisms. Literature reported that ER stress can effectively induce autophagy in cells because malignant tumor cells need to re-use their organelles to maintain growth (Muñoz-Guardiola et al., 2020). Another important finding of the current study is the involvement of ER stress in autophagy to monitor CRC growth process (Figure 8). Above results are consistent with clinical data from CRC patients. In summary, the current study revealed the anti-cancer roles of CPT in colorectal cancer, which is mediated via autophagy signaling and ER stress. CPT is a promising therapeutic candidate for CRC treatment.

## Data Availability

The raw data supporting the conclusion of this article will be made available by the authors, without undue reservation.

## Abbreviations

ATF6, activating transcription factor-6; ATG, autophagy-related gene; AO, Acridine orange; BIP, immunoglobulin heavy chain binding protein in pre-B cells; BSA, bovine serum albumin; CPT, cryptotanshinone; CRC, colorectal cancer; Cis, cisplatin; ER stress, endoplasmic reticulum stress; DMSO, dimethyl sulfoxide; LDH, lactate dehydrogenase; IHC, immunohistochemistry; IRE1α, inositol-requiring enzyme 1α; 4-PBA, sodium 4-phenylbutyrate; PKR, protein kinase RNA; PERK, protein kinase RNA (PKR)-like ER kinase; UPR, unfolded protein response
